# Relevant factors of self-care in children and adolescents with spastic cerebral palsy

**DOI:** 10.1371/journal.pone.0254899

**Published:** 2021-07-21

**Authors:** Yasuaki Kusumoto, Kenji Takaki, Tadamitsu Matsuda, Osamu Nitta

**Affiliations:** 1 Department of Physical Therapy, Fukushima Medical University School of Health Sciences, Fukushima city, Fukushima, JP; 2 Faculty of Health Sciences, Department of Physical Therapy, Tokyo University of Technology, Ohta-ku, Tokyo, JP; 3 Faculty of Health Sciences, Department of Physical Therapy, Juntendo University, Bunkyo-ku, Tokyo, JP; 4 Faculty of Health Sciences, Department of Physical Therapy, Tokyo Metropolitan University, Arakawa-ku, Tokyo, JP; Universitat de les Illes Balears, SPAIN

## Abstract

**Objective:**

Manual ability is considered one of the factors that can predict functional independence in activities of daily living. For evaluating personal tasks such as self-care, the Pediatric Evaluation of Disability Inventory (PEDI) comprises/introduces/offers a set of useful measures that assist in enhancing the capability for self-care among children and adolescents with cerebral palsy (CP). The aim of this study was to investigate the relevant factors of self-care capability and performance in children and adolescents with spastic CP.

**Methods:**

This was a cross-sectional study. Seventy-six children and adolescents with spastic CP (between 5 and 18 years of age), representing levels I to IV of the Gross Motor Function Classification System-Expanded & Revised version (GMFCS), were analyzed. Multiple linear regression analysis with forward stepwise selection was conducted to examine which determinants were related to self-care capability and performance. Independent variables were age, CP type, GMFCS, Manual Ability Classification System, Box and Block Test, and grip strength in the dominant and non-dominant hands. Dependent variables were scores for the PEDI Functional Skills Scale and the PEDI Caregiver Assistance Scale.

**Results:**

Results of the multiple regression analysis showed that the PEDI Functional Skills scale scores were correlated with the Box and Block Test in the dominant hand and GMFCS (Adjusted R^2^ = 0.69). The PEDI Caregiver Assistance Scale scores were correlated with the Box and Block Test in the dominant hand, GMFCS, and age (adjusted R^2^ = 0.71).

**Conclusion:**

When considering self-care of children and adolescents with spastic CP, it is necessary to consider the evaluation of upper limb dysfunction in addition to GMFCS.

## Introduction

Children with cerebral palsy (CP) present with various upper extremity impairments, including muscle weakness, impaired motor fluency, accuracy, dexterity, and adaptability to the environment [1,2]. Impaired manual ability is characterized by impairments in range of motion, grip strength, and motor control [3]. Children with CP often have difficulty grasping, releasing, and manipulating objects—all skills needed for daily living activities [4]. Many children can efficiently perform activities of daily living (ADL) with one hand, but most manual ADL are easier to perform using both hands. Many upper limb assessment tools have been confirmed to be reliable and valid for the measurement of functional skills in children with CP [2]. Dominant hand (DH) performance of activities requiring manual dexterity is better than non-dominant hand (NDH) performance in children with CP; this applies to both fine and gross manual dexterity [5]. Manual ability should be specifically evaluated given its importance in the daily activities of children and adolescents [6].

In a study by Arnould et al, three motor impairments (grip strength, gross manual dexterity, and fine finger dexterity) and three sensory impairments (tactile pressure, detection, and stereognosis, and proprioception) were evaluated to investigate the relationship between hand impairments and manual ability. Results showed that gross manual dexterity in the DH and grip strength in the NDH were the best independent predictors of manual ability [5]. In another study, gross manual dexterity and grip strength in both hands were the strongest predictors of manual ability [7]. Therefore, assessment of gross manual dexterity and grip strength are important when considering upper limb impairments. Previous researchers have also stated that the relationships between hand function (as measured by the Manual Ability Classification System [MACS]) and self-care activities are significant [8–10]. Manual ability can predict functional independence in ADL. Previous studies have also reported that gross motor abilities are strongly associated with everyday functioning in children with CP [11–13]. In particular, the Gross Motor Function Classification System (GMFCS) has been found to be predictive of self-care [9]. Clinical experience shows that hand function—which is affected by the degree of deformity, spasticity, sensory deficit, and motor control—is also important for self-care. Previous studies in Asia have reported that the MACS and GMFCS correlate with levels of functioning in ADL, instrumental ADL, and social participation in children with CP [14,15]. However, these methods do not provide a full assessment across various life situations [16]. Limited hand function is common in all types of CP, but the specific characteristics of this limitation varies considerably between different CP subtypes [17]. CP type can be classified by both functional and traditional classification systems and by using topographic and physiological classifications [18]. Topographic classification does not consider functional abilities, but it has been used to investigate the relationship between ADL and the localization/limb distribution of neuromotor impairment in spastic CP [5,7,14,15].

ADL include main occupational activities or participation in activities across different environments (home, school, community, etc.). ADL are conceptualized in the “Activities and Participation” domain of the International Classification of Functioning, Disability and Health (ICF) and are defined as life tasks required for self-care and self-maintenance, such as dressing, bathing, and eating [19]. There are many tools useful for evaluating level of functioning in self-care tasks. The Pediatric Evaluation of Disability Inventory (PEDI) is a measure of ADL capability among children aged 6 months to 7 years and is used to report functional skills in children and adolescents with CP [20]. PEDI evaluates both common parent report outcome measures and client-based measures [20–22]. However, PEDI has many components and is often difficult to administer in busy clinical settings. The PEDI-Computer Adaptive Test (CAT) is a newly adapted PEDI assessment for children and adolescents from birth to 20 years of age that was developed based on formal, experiential data collected on the original PEDI [23,24]. Although PEDI has been translated into many languages and is used worldwide, the translations of PEDI-CAT are few, and the number of countries it can be used in remains limited.

Goal-directed activity-focused physiotherapy, hand-arm bimanual intensive therapy, and constraint-induced movement therapy are being used to improve self-care in children with CP [25–27]. Children with CP usually show hand dominance on the less affected side; it is important to identify factors, such as this, that affect self-care activities so that interventions can be appropriately designed and tailored to the individual. Furthermore, the identification of these relevant self-care factors will lead to a greater understanding of the condition overall. Therefore, when considering ADL independence in children and adolescents with CP, it is necessary to consider upper limb function, gross motor ability, and CP type. Accordingly, the aim of the present study was to investigate the factors that influence self-care capability and performance in children and adolescents with spastic CP.

## Materials and methods

### Participants

Children and adolescents with CP were recruited from a hospital and three medical centers in neighboring Tokyo (Minamitama Orthopedic Hospital and Shimada Ryoiku Center Hachiouji) and Kanagawa (Seiyo Gakuen and Taiyounomon Welfare Medical Center) between June 2015 and May 2016. The inclusion criteria were as follows: (1) a diagnosis of spastic CP; (2) age 5−18 years; and (3) ability to communicate and follow instructions (levels I-III on the Communication Function Classification system). The exclusion criteria were as follows: (1) GMFCS level V and (2) history of orthopedic surgery or botulinum toxin use within the previous 6 months. In the preliminary study, most of the children at GMFCS level V could not perform the Box and Blocks test and grip strength test. Therefore, children whose GMFCS level was V were excluded from the present study.

Eighty one patients were referred to this study in response to posters and pamphlets that were distributed. Of the 81 patients, 76 participants and/or their parents provided informed consent. The study population consisted of 76 children and adolescents with CP (average age: 13.6 ± 3.7 years). Forty were boys (52.6%), and 36 were girls (47.4%) (Table 1). The types of CP represented by the participants were as follows: spastic hemiplegia in 11 (14.5%), spastic diplegia in 45 (59.2%), and spastic quadriplegia in 20 (26.3%). The participants of this study were considered to be representative of the Japanese population as the breakdown of the CP type was similar to previous studies [28].

**Table 1:** Attributes of the participants and the measurement results.

Age, years (range)	13.6 ± 3.7 (5–18)
Body mass index	18.6 ± 3.2
CP Type (hemiplegia, diplegia, quadriplegia), n	11, 45, 20
GMFCS (I, II, III, IV), n	22, 21, 13, 20
MACS (I, II, III, IV, V), n	33, 21, 13, 7, 2
Scaled score PEDI-FSS, score	79.4 ± 18.2
Scaled score PEDI-CAS, score	80.7 ± 23.6
BBT DH, score	38.0 ± 16.7
NDH, score	29.8 ± 17.8
Grip strength DH, kg	18.2 ± 10.7
NDH, kg	13.8 ± 10.4

GMFCS: Gross Motor Function Classification System; MACS: Manual Ability Classification System; PEDI-FSS: Pediatric evaluation of disability inventory functional skills scales; CAS: Caregiver assistance scale; BBT: Box and block test; DH: Dominant hand; NDH: Non-dominant hand.

The children were included in the study after their parents signed the informed consent form. This study was approved by the Tokyo University of Technology of Health Sciences Ethical Review Board (authorization number: E14HS-004).

### Design

This was a cross-sectional study of children and adolescents with spastic CP.

### Measures

The GMFCS and MACS levels were established based on the therapist’s observations of the participant’s behavior. The GMFCS was used to classify gross motor skills. The MACS was used to classify the manual abilities. These are five-level classification systems designed to represent a child’s abilities and limitations in gross motor function and manual abilities. Level I indicates minimal or no disability, while level V indicates complete dependence on external assistance for mobility [29] and hand use [10]. The Japanese version of the GMFCS used in this study reportedly has good reliability for children with CP [30]. The Japanese version of the MACS used in this study also has good reliability and validity in children with CP [31].

The following three clinical tools were used in this study: the PEDI, Box and Blocks test, and grip strength test. Trained pediatric physical therapists and occupational therapists administered the assessments. The self-care domain of the PEDI was administered by trained physical therapists and occupational therapists through a structured interview with the parents.

#### Pediatric Evaluation of Disability Inventory (PEDI)

The PEDI was used to measure self-care capability and performance. Many studies have demonstrated the PEDI to have excellent reliability, validity, and responsiveness [20]. The PEDI is used to assess children’s capabilities and performance in three domains: self-care, mobility, and social functioning. The PEDI Functional Skills Scale (PEDI-FSS) is a 193-item inventory that assesses an individual’s potential abilities. The response for each item is either “capable” (score = 1) or “incapable” (score = 0). The PEDI Caregiver Assistance Scale (PEDI-CAS) is a 6-point ordinal scale that assesses an individual’s actual abilities in the context of his or her daily environment (home, school, etc.). In this study, we used a validated Japanese version of the PEDI for an interview with the parent and only scaled the scores for the PEDI-FSS and PEDI-CAS of the self-care domains on the PEDI. A Japanese version of the PEDI manual was published in 2003 and this tool has good reliability and validity for measuring self-care capability and performance in children with CP [32].

#### Box and Blocks test (BBT)

The BBT was used to evaluate gross manual dexterity. The test-retest reliability of the BBT was reported to be between 0.93 and 1.00 [33]. Participants were instructed to grasp a block from one compartment of the box, transport it over the partition, and release it into the opposite compartment of the box as quickly as possible within 60 s. They performed the test once with each hand, starting with the DH [34].

#### Grip strength

Grip strength was measured using a method recommended by the Japanese Ministry of Education, Culture, Sports, Science, and Technology [35]. Grip strength was determined as the maximum force exerted on a hand dynamometer (TOEI LIGHT, Grip D, Japan) across two trials. Grip strength was assessed for both hands, starting with the DH.

### Statistical analysis

The normality of all data was initially confirmed using the Shapiro-Wilk test. To examine which of the determinants were related to self-care capability and performance, multiple linear regression analysis with forward stepwise selection was performed with age, CP type, GMFCS, MACS, BBT, and grip strength in the DH and NDH as independent variables, and PEDI-FSS and PEDI-CAS scores as dependent variables. In conducting multiple linear regression analysis, the relationship between the dependent and independent variables was determined beforehand using a single regression analysis. Multicollinearity was confirmed using the variance inflation factor, and model fit was examined using the coefficient of determination (R^2^). All analyses were performed using the SPSS statistical package for Windows, version 21.0. Statistical significance was set at p <0.05.

## Results

The attributes of the participants and the measurement results are shown in Table 1. The results of the correlation analyses between the independent variables are presented in Table 2. None of the independent variables had absolute correlation coefficient values greater than 0.9. The results of multiple regression analysis are presented in Table 3. The variance inflation factor of the obtained variables was less than 10, and no multicollinearity was present. The PEDI-FSS scores were correlated with BBT (DH) scores and GMFCS (adjusted R^2^ = 0.69). PEDI-CAS scores were correlated with BBT (DH) scores, GMFCS, and age (adjusted R^2^ = 0.71).

**Table 2:** Results of the correlation analysis between independent variables.

	age	CP Type	GMFCS	MACS	BBT DH	BBT NDH	Grip strength DH
CP Type	0.21						
GMFCS	0.08	0.69 *					
MACS	0.01	0.67 *	0.78 *				
BBT DH	0.09	-0.57 *	-0.69 *	-0.78 *			
BBT NDH	0.13	-0.41 *	-0.50 *	-0.73 *	0.82 *		
Grip strength DH	0.47 *	-0.25 †	-0.33 *	-0.45 *	0.62 *	0.56 *	
Grip strength NDH	0.50 *	-0.13	-0.29 †	-0.48 *	0.61 *	0.78 *	0.76 *

* p<0.01

† p<0.05, analysis by Spearman’s rank correlation coefficient, and Pearson’s correlation coefficient, GMFCS: Gross Motor Function Classification System; MACS: Manual Ability Classification System; BBT: Box and block test; DH: Dominant hand; NDH: Non-dominant hand.

**Table 3:** Results of the multiple regression analyses for the PEDI-FSS and the PEDI-CAS.

		Partial regression coefficient	Standard partial regression coefficient	Variance inflation factor	*p* value
PEDI-FSS	Constant	64.800			
	BBT DH	0.658	0.605	1.952	p<0.01
	GMFCS	-4.221	-0.287	1.952	p<0.01
PEDI-CAS	Constant	52.271			
	BBT DH	0.692	0.491	2.072	p<0.01
	GMFCS	-7.386	-0.388	2.080	p<0.01
	Age	1.480	0.235	1.075	p<0.01

R^2^ of PEDI-FSS is 0.69. R^2^ of PEDI-CAS is 0.71.

PEDI-FSS: Pediatric evaluation of disability inventory functional skills scales; CAS: Caregiver assistance scale; BBT: Box and block test; DH: Dominant hand; GMFCS: Gross Motor Function Classification System.

## Discussion

PEDI can measure self-care capability through PEDI-FSS and the performance of self-care through PEDI-CAS. These tools encompass activities that are important in the ICF concept for children and adolescents with CP [20]. In this study, BBT(DH) and GMFCS were identified as factors related to both the capability and performance of self-care. As shown in Table 2, BBT (DH) had a high positive correlation with BBT (NDH). Grip strength in both hands was negatively correlated with CP type, GMFCS, and MACS. Additionally, age was identified as a relevant factor for PEDI-CAS, which is related to self-care performance. According to CP type, MACS level, and grip strength, differences in PEDI-self-care scores were not statistically significant.

The PEDI self-care domain includes factors such as hair brushing, tooth brushing, washing of the body and face, dressing, and use of utensils and drinking containers. Gross manual dexterity in the DH is required for almost all these self-care activities. Since there is a positive correlation between BBT (DH) and grip strength, there may be a need to consider the importance of grip strength during individual rehabilitation for self-care in children with cerebral palsy. However, the present study did not analyze in detail the relationships among the various components of the PEDI self-care domain. Further studies should perform a detailed examination of each component, such as brushing and dressing.

The PEDI self-care domain itself is not directly related to mobility. In a previous study, GMFCS levels and PEDI mobility were factors that significantly influenced PEDI self-care in children with CP [36]. Moreover, another study using multivariate analysis indicated that GMFCS and intellectual capacity were the strongest determinants of the development of self-care activities in children with spastic bilateral and unilateral CP [37]. Findings from these previous studies and our results may be supported by other studies that suggest that gross motor capacity is strongly associated with everyday functioning [11–13]. The present study demonstrates that both gross manual dexterity in the DH and gross motor function are good predictors of self-care.

Previous studies have stated that the relationship between MACS and self-care activities is significant [8–10]. In the present study, hand function was shown by the BBT (DH) and not by the MACS. The results of the previous studies were different from ours possibly because only severe classifications of gross motor and manual ability were measured in previous studies; they did not consider the functional impairments in gross manual dexterity and grip strength, which were measured in this present study. The performance of dexterity tasks requires gross and fine hand motions and coordination [38]. Dexterity tasks are generally performed over a short period of time. Manual ability, such as that measured by MACS, may reflect daily activities performed continuously throughout the day [7]. Therefore, when considering self-care of children and adolescents with CP, it is necessary to consider the evaluation of upper limb dysfunction in addition to MACS.

Sitting balance and gross manual dexterity are related [39,40]. Postural stability and manual dexterity are related to each other [41], and postural stability and motor skills such as GMFCS are likewise related [42]. As improved proximal stability achieved through better trunk control may lead to improvements in upper extremity function [41,43], it may be necessary to focus on trunk impairment to improve self-care in children and adolescents with spastic CP.

CP type was not a significant factor in the current study. When the CP types are classified as spastic unilateral and bilateral, the MACS is a strongest predictor of PEDI self-care skills [9]. Moreover, self-care in hemiparetic CP and quadriparetic CP is more strongly related to MACS than to GMFCS [10]. In a previous study that included patients with spastic hemiplegia, spastic diplegia, spastic quadriplegia, dyskinetic, and mixed type, self-care PEDI-FSS scores were predicted by CP type, learning difficulties, age, and selective motor control (adjusted R^2^ = 0.73) [13]. These results may indicate that the relationship to the "self-care of PEDI" varies depending on how the CP type is classified and the number of CP types in each analysis. In the future, it may be important to consider the types of CP classifications, such as traditional classifications and the Surveillance for Cerebral Palsy in Europe classification.

Lack of self-care can lead to significant limitations on participation in community life [20]. Since age was related to the PEDI-CAS, rehabilitation should be implemented to ensure continuous improvement in self-care from childhood to adulthood. Task-oriented approaches to the treatment of the affected hand improve functional activities and basic daily activities of patients with spastic hemiplegia due to CP [44,45]. These activities require manual dexterity, fine motor performance, and grip strength. When designing a task-oriented approach to improve self-care, it may be necessary to consider manual dexterity.

## Limitations

This study has some limitations. This was a cross-sectional study with a small number of participants in the spastic hemiplegia group, so generalization of the results may be limited. Because GMFCS level V children were excluded from the present study, self-care in children with spastic cerebral palsy of all motor levels has not been accurately measured. Upper limb function of children with GMFCS level V should be considered in future studies. Degree of deformity, spasticity, and motor control affect hand function and ADL independence in individuals with CP [46,47]. Spasticity was not independently measured in this study, and grip strength was measured as the maximum force exerted when both spasticity and voluntary muscles were engaged. Therefore, it was not possible to clearly separate spasticity and voluntary muscle use in grip strength measurements. In this study, only classification systems and clinical tools that were relevant in previous studies were examined; therefore, it is unclear how results from different assessment tools might affect outcomes related to self-care. Future studies should include relevant factors, such as trunk function and other dysfunctions.

## Conclusions

This study investigated the relevant factors associated with self-care capability and performance in children and adolescents with spastic CP. This study supports the use of various classification systems and assessments in clinical practice as they are integral to clinical success. The results showed that gross manual dexterity in the DH and gross motor abilities were important predictors of self-care capability in children and adolescents with spastic CP. Gross manual dexterity in the DH, gross motor abilities, and age were important predictors of self-care performance in children and adolescents with spastic CP.

## Supporting information

S1 Data (XLSX)
